# BIG LITTLE THIEFS - Kleptomania Treatment

**DOI:** 10.1192/j.eurpsy.2022.2279

**Published:** 2022-09-01

**Authors:** S. Mouta, J. Correia, I. Fonseca Vaz, S. Freitas Ramos, B. Jesus, S. Fontes

**Affiliations:** Unidade Local de Saúde da Guarda, Departamento De Psiquiatria E Saúde Mental, Guarda, Portugal

**Keywords:** Kleptomania, Psychotherapy, Electroconvulsive therapy, Pharmacotherapy

## Abstract

**Introduction:**

Kleptomania is characterized by recurrent failure to resist the impulse to steal items of little value despite the ego-dystonic impulse and awareness of the wrongfulness of the act. Its prevalence is considered to be 0.6–0.8% in the general population and it is mostly comorbid with other psychiatric disorders. Kleptomania is a disabling disorder since patients suffer from emotional distress and impaired functioning.

**Objectives:**

Although there is no cure, treatment may help prevent Kleptomania worsening and its negative consequences. We propose a review of the therapeutic approach to this disease.

**Methods:**

Non-systematic literature review.

**Results:**

No effective treatment is available for Kleptomania. Better efficacy can be achieved by combining psychotherapy with pharmacotherapy. Different treatment interventions can be selected based on clinical similarities to other disorders, co-occurring conditions or behavioral core features. Patients with significant mood symptoms may benefit from mood stabilizers or antidepressants. For patients with shoplift cravings and/or family history of substance use disorders, Naltrexone may reduce symptoms. Stimulants may be useful for Kleptomania bassociated with Attention Deficit Hyperactivity Disorder impulsivity. Benzodiazepines are effective in tension relief when used as adjuvants, at the beginning of treatment. Electroconvulsive therapy should be reserved for patients with treatment-resistant symptoms and comorbid depression. Cognitive-behavioral therapy has replaced Psychoanalytic and Psychodynamic psychotherapies.

**Conclusions:**

Treatment helps decrease disruption to the person’s life, preventing the intense shame, legal, social, family, and occupational repercussions of Kleptomania. Although pharmaceutical and psychosocial interventions are available, we still lack specific treatments for Kleptomania.

**Disclosure:**

No significant relationships.

